# The Origin of Life: Models and Data

**DOI:** 10.1007/s00239-017-9783-y

**Published:** 2017-02-27

**Authors:** Kathryn A. Lanier, Loren Dean Williams

**Affiliations:** School of Chemistry and Biochemistry, Georgia Institute of Technology, Atlanta, GA 30332-0400 USA

**Keywords:** Origins of life, Molecular toolbox of Life, Translation, RNA world, Metabolism world, Clay world, Thermal vent world, Membrane world

## Abstract

A general framework for conventional models of the origin of life (OOL) is the specification of a ‘privileged function.’ A privileged function is an extant biological function that is excised from its biological context, elevated in importance over other functions, and transported back in time to a primitive chemical or geological environment. In RNA or Clay Worlds, the privileged function is replication. In Metabolism-First Worlds, the privileged function is metabolism. In Thermal Vent Worlds, the privileged function is energy harvesting from chemical gradients. In Membrane Worlds, the privileged function is compartmentalization. In evaluating these models, we consider the contents and properties of the Universal Gene Set of life, which is the set of orthologous genes conserved throughout the tree of life and found in every living system. We also consider the components and properties of the Molecular Toolbox of Life, which contains twenty amino acids, eight nucleotides, glucose, polypeptide, polynucleotide, and several other components. OOL models based on privileged functions necessarily depend on “takeovers” to transition from previous genetic and catalytic systems to the extant DNA/RNA/protein system, requiring replacement of one Molecular Toolbox with another and of one Universal Gene Set with another. The observed robustness and contents of the Toolbox of Life and the Universal Gene Set over the last 3.7 billion years are thought to be post hoc phenomena. Once the takeover processes are acknowledged and are reasonably considered, the privileged function models are seen to be extremely complex with low predictive power. These models require indeterminacy and plasticity of biological and chemical processes.

## OOL Models

A broad array of origin of life (OOL) models are described in the current literature. However, the diversity of these models is superficial. In fact, many OOL models use the same general framework and are governed by common precepts. A primary theme of many OOL models is the specification of one, or in some cases two, ‘privileged function(s).’ Privileged functions are extant biological functions that are considered by OOL model builders to be so essential and fundamental to life that they must also be a requisite for the origin of life. In a given OOL model, a defining privileged function is excised from its biological context, elevated in importance over other biological functions and reassigned in space, time and environment. The privileged function is shifted to geological antiquity, to a date preceding the onset of other biological functions, and is transported to a primitive chemical or geological environment. OOL models are defined and distinguished by their privileged function(s).

The primary appeal of privileged function models is the appearance of simplicity. In comparison to real biological or real abiotic systems, artificial constructs with one or a few privileged functions grant few variables, contain few components, and exhibit few dependencies.

**Fig. 1:**
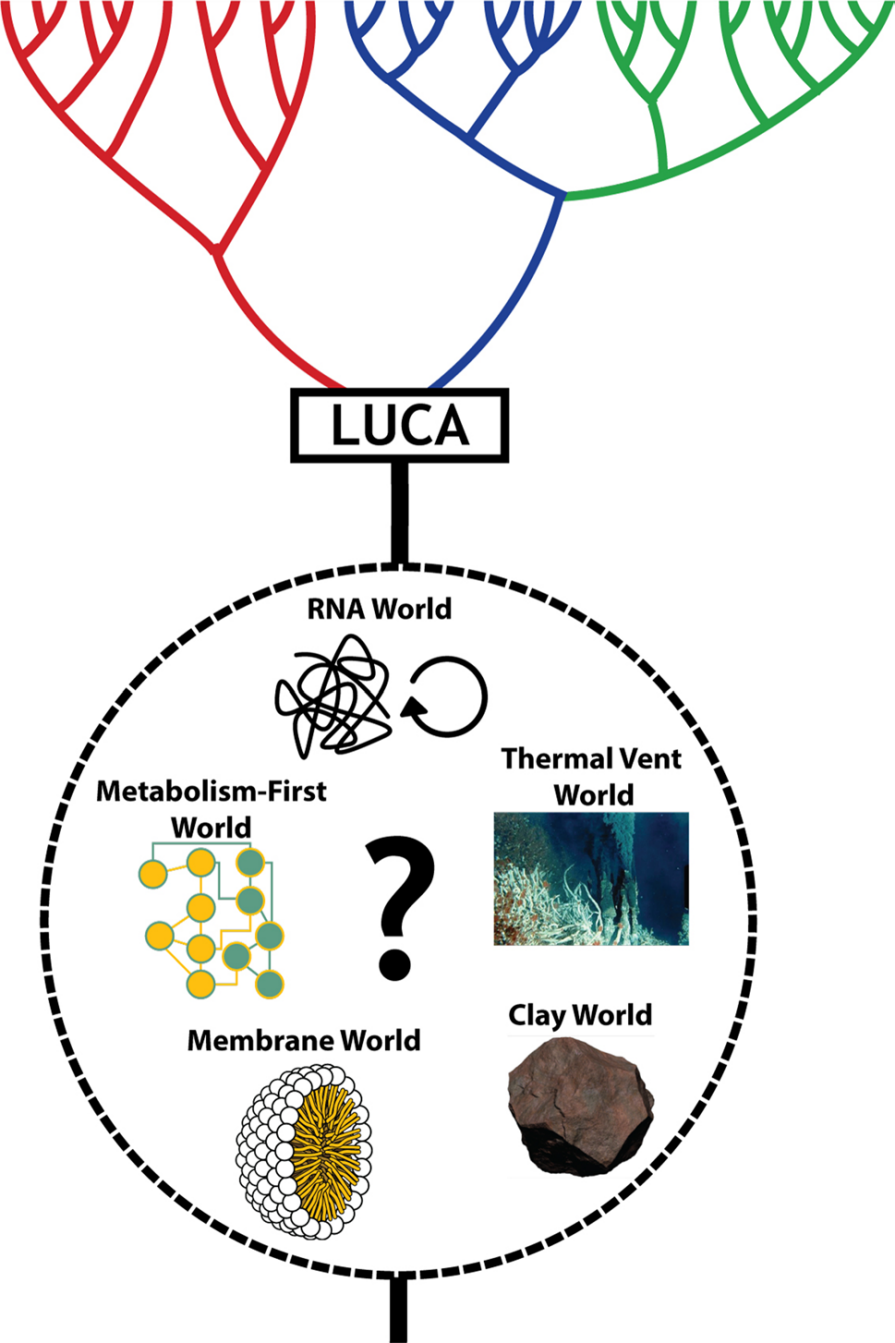
Schematic of the universal tree of life illustrating privileged functions in various OOL models. Each model leads to the last universal common ancestor (LUCA)

Here, we give brief introductions to OOL models and identify their privileged functions (Fig. 1). We formulate general principles for evaluating these models. However, the term ‘model’ requires qualification in this context. Conventional scientific models are falsifiable. OOL models are not. OOL models are not characterized by or linked to experiments, either proposed or performed, that would lead to rejection of the models. Alternatives to the classification system used here for OOL models do not change our overall conclusions.

### RNA Worlds

In RNA Worlds, the privileged function is replication (Rich 1962; Crick 1968; Orgel 1968; Miller and Orgel 1974; Gilbert 1986; Benner et al. 1989; Cech 2009; Neveu et al. 2013; Higgs and Lehman 2015). RNA is the template, product, and mediator of replicative processes. The defining component of an RNA World is a ribozyme RNA polymerase. In this class of models, a ribozyme polymerase kick-started Darwinian evolution prior to or early in life on Earth. As stated by Orgel, “the origin of RNA replication is … the central puzzle of the origin of life” (Orgel 1995). Early life is defined by an RNA-based Darwinian system that uses RNA exclusively or predominantly for both information storage and catalysis. Biology subsequently experienced one or more phase changes (catalytic and genetic takeovers), when translation systems began producing coded protein for catalysis and when DNA was adopted for information storage. In RNA World models, the chemical instability and inferior catalytic capabilities of RNA drove a ‘catalytic takeover’ (from RNA to protein) and a ‘genetic takeover’ (from RNA to DNA).

### Clay Worlds

In Clay Worlds, as in RNA Worlds, the privileged function is replication. However, in Clay Worlds, replication was initiated by minerals (Cairns-Smith 1990, 2008; Yang et al. 2013). Cairns-Smith proposed a Darwinian system in which the initial templates and products of the replicative process are mineral. By dissolution and precipitation, clays acted as genetic materials that also formed templates for organic molecules. Ferris proposed that polymerization of organic informational molecules was catalyzed by clay surfaces (Ferris 2002; Aldersley et al. 2011). Ultimately organic polymers were released from the nurturing mineral surface, possibly by changes in geochemical conditions. A genetic takeover caused a transition from mineral templates to the extant biological world of nucleic acid-based genetics.

### Metabolism-First Worlds

In Metabolism-First models, the privileged function is obviously metabolism (Dyson 1982, 1999; Wächtershäuser 1988; Wachtershauser 1988; Shapiro 2007). In these models, autocatalytic networks of self-sustaining chemical reactions, in the absence of replicative or catalytic organic polymers, extracted and captured energy from the environment. Wächtershäuser, for example, proposed that iron sulfide minerals catalyzed chemical reactions in the form of a Krebs-like cycle running in reverse. These reactions were said to form amino acids, nucleotides, and lipids. Protein enzymes replaced mineral catalysts in a catalytic takeover, and polymer-based replicative systems were acquired subsequently. Proteins and nucleic acids are the evolutionary outcome of mineral-catalyzed metabolism. In this model, extant metabolism based on protein enzymes was introduced after organic molecules polymerized to act as biological catalysts for the synthesis of additional organic molecules.

### Thermal Vent Worlds

In Thermal Vent Worlds, which are a subset of Metabolism-First Worlds, the privileged function is the harvesting of energy from chemical gradients (Martin et al. 2008; Sojo et al. 2016). Submarine hydrothermal vents, which currently harbor rich biological communities, are proposed to have abiotically synthesized simple organic molecules followed by polymers (Corliss et al. 1981). Thermal vents cause chemical gradients at interfaces between hydrothermal effluents and bulk ocean water. It is proposed that gradients across mineral barriers were converted via catalytic takeover into biological chemiosmotic systems that harvest energy from membrane gradients. Support for this model is seen by some in parallels between proposed redox chemistry in hydrothermal systems and metabolic reactions in prokaryotes.

### Membrane Worlds

In Membrane Worlds, the privileged function is compartmentalization (Ourisson and Nakatani 1994; Segré et al. 2001; Szostak et al. 2001; Koonin and Martin 2005). In some versions of these models, abiotic amphipaths such as terpenoids self-assembled to form vesicles. The formation of compartments is proposed to be highly consequential, causing cascades from simple to complex systems. In several OOL models, compartmentalization is a secondary privileged function, used as an adjunct to a primary privileged function.

## The Data

### Data Filters

There is a wealth of data that can help one understand the ancient earth and primitive biology. As noted above, numerous OOL models are available to which one can attempt to fit the data. However, it is important that data come before models; affection for a model should not cause data to be disregarded or cherry-picked. Using phylogenetic and biochemical reasoning, Gogarten and others have concluded that LUCA was a prokaryotic-like terrestrial life form, using DNA as genetic material, RNA as message, ribosomes for coded synthesis of proteins from 20 amino acids, with membranes and chemiosmotic coupling (Zhaxybayeva and Gogarten 2004; Peretó et al. 2004; Gogarten and Deamer 2016). Martin uses phylogenetic reasoning to conclude by contrast that LUCA was dependent upon geochemistry of hydrothermal vents and was only “half-alive” (Weiss et al. 2016). The contradictions in these conclusions suggest that approximations, computation short-cuts, model-dependent inferences, and biases interfere with interpretation of data. Our goal here is to enumerate and define types of data that we believe are useful for understanding and evaluating OOL models. We have developed several filters by which we weigh different types of data.

The most significant data are derived from authentic measurements made on actual biological or abiotic systems. The most important data has authenticity that does not depend on speculative models and significance that does not depend on indirect inference. Useful data are found in the rich abiotic chemical inventory in carbonaceous chondrite meteorites (Martins et al. 2008; Schmitt-Kopplin et al. 2014) and in the comparison of that inventory with an uninteresting inventory of hydrothermal vents (McCollom and Seewald 2007; Proskurowski et al. 2008; Lang et al. 2010; McDermott et al. 2015). In fact, the seminal hydrothermal vent model of the OOL (Corliss et al. 1981) has been directly falsified by demonstrations that vents do not produce the molecules proposed in the model. Other examples of useful data are sequences and structures of ribosomal proteins and ribosomal RNAs (Woese and Fox 1977; Ramakrishnan 2002; Steitz 2008), and the statistics of their similarity and distribution over phylogeny (Fournier and Gogarten 2010).

Data are less useful when relevance and significance are model dependent and/or when derived from arbitrary experimental conditions. A specific example of data with low utility for understanding the OOL is the observation of and properties of an *in vitro* selected ribozyme that catalyzes a Diels–Alder reaction (Seelig and Jaschke 1999). The authors make the reasonable claim that their Diels–Alder ribozyme merely reveals the potential of small ribozymes for catalyzing organic transformations. In our view, this ribozyme is not relevant to the OOL because of the following reasons:

1. no evidence for Diels–Alder ribozymes has been found among extant biological structures or sequences,
2. no evidence for Diels–Alder ribozymes has been found in ancestral biological systems,
3. the processes and reagents used to obtain the Diels–Alder ribozyme are inconsistent with plausible early biological or abiotic environments; the *in vitro* selection process employed modern protein enzymes such as polymerases and reverse transcriptases and is not representative of pre-protein RNA World environments, and
4. thus far, a null hypothesis has not been evaluated, in which alternative polymers such as polysaccharides would be investigated for ability to catalyze Diels–Alder reactions.

Factors that constrain the significance of a Diels–Alder ribozyme for understanding the OOL apply equally to other in vitro selected ribozymes, including RNA polymerase ribozymes (Mutschler et al. 2015; Horning and Joyce 2016). The in vitro selection of RNAs, using modern methods of molecular biology, does not in our view provide important information relevant to the OOL.

### LUCA and the Universal Gene Set

We consider the contents and properties of the Universal Gene Set of life, which is the set of genes shared as orthologs throughout the tree of life, and found in essentially every living system, to be important and useful data. The size and composition of the Universal Gene Set are generally agreed upon (Koonin 2003; Harris et al. 2003; Charlebois and Doolittle 2004). In some highly dependent symbionts, components of the Universal Gene Set might be absent from a given species.

**Fig. 2:**
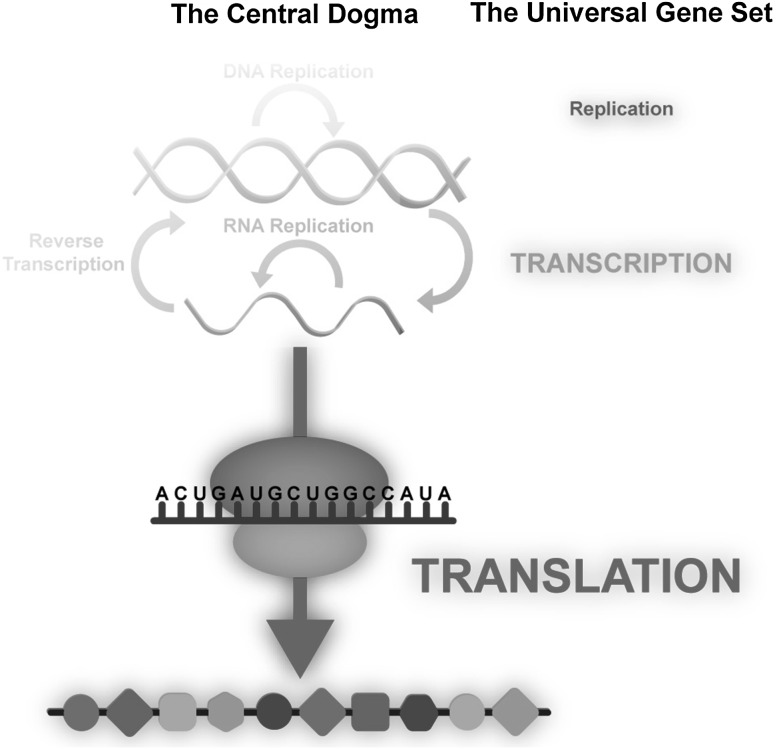
The central dogma of molecular biology, emphasizing the contents of Universal Gene Set of life, which includes genes for ribosomal RNAs, ribosomal proteins, aminoacyl tRNA synthetases and translation factors

The Universal Gene Set is small and distinctly non-random. Koonin’s version of the Universal Gene Set, for example, contains around 65 genes. Fifty-three universal genes are directly involved in translation. These include genes for ribosomal RNAs, ribosomal proteins, aminoacyl tRNA synthetases, and translation factors (Fig. 2). A few members of the Universal Gene Set are involved in transcription and even fewer in replication. The Pace and Doolittle versions are very similar to the Koonin Universal Gene Set.

The Universal Gene Set is the most robust and unchanging subset of the gene set of LUCA. As noted above, there is evidence to suggest that LUCA contained genes beyond the Universal Gene Set (Zhaxybayeva and Gogarten 2004; Peretó et al. 2004; Gogarten and Deamer 2016). Specifically, for several proteins involved in DNA replication, ancestry at LUCA is indicated by conservation of three-dimensional structures, even though sequences are not conserved (Edgell and Doolittle 1997). In addition, LUCA may not have been a single entity. Genes that are ancestral to the Universal Gene Set may have been hosted in a variety of types of organisms (Zhaxybayeva and Gogarten 2004).

### LUCA and the Molecular Toolbox of Life

**Fig. 3:**
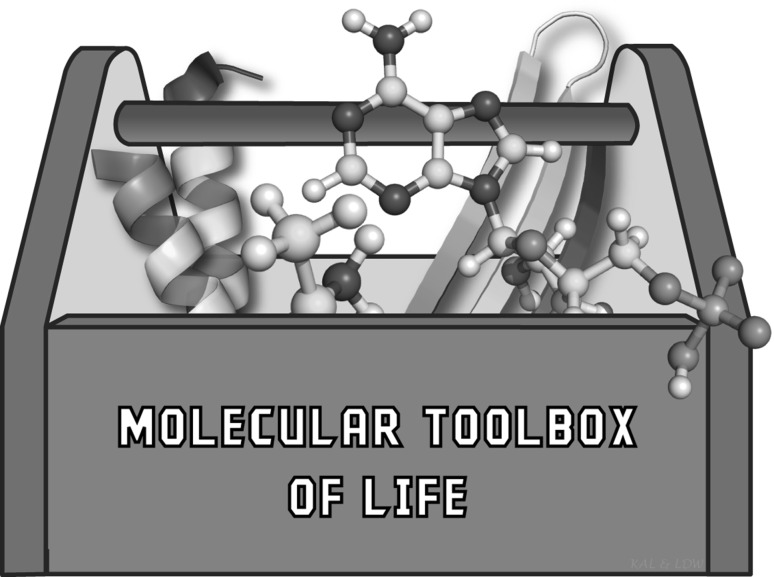
Schematic of the molecular toolbox of life, which contains the small molecules and macromolecular backbones and motifs that are universal to all living systems. This image was inspired by Jacob (1977)

We consider the components and properties of the Molecular Toolbox of Life (Fig. 3) (Jacob 1977) to be another source of important and useful data. Biological systems, regardless of domain or environment, use a common set of molecular components that is fixed over time and is surprisingly restricted in composition. The universal molecules of life are composed of twenty amino acids, eight nucleotides, glucose, S-adenosylmethionine, coenzyme A, nicotinamide adenine dinucleotide, and several other components. Also, universal to life are several polymer backbone types, including polypeptide, polyribonucleotide, and polydeoxyribonucleotide. The diverse morphology of eukaryotes, from algae to whales, and the diverse metabolism of prokaryotes, from methanogenic archaea to sulfur oxidizing bacteria, are all built with the same small toolbox of organic molecules (Jacob 1977). Diverse organisms are distinguished not by differences in composition of their Molecular Toolboxes but by differences in organization of components of a common Molecular Toolbox.

### Persistence and Robustness

A small set of organic molecules and genes are found in everything alive, in all bacteria, archaea, and eukaryote. If LUCA was prokaryote-like (Zhaxybayeva and Gogarten 2004; Peretó et al. 2004; Gogarten and Deamer 2016), then the Toolbox of Life and Universal Gene Set have been fixed since LUCA, which is thought to have existed over 3.7 billion years ago (Doolittle 2000; Nutman et al. 2016). There are no direct data to support the hypothesis that alterations of the Molecular Toolbox and the Universal Gene Set are allowed by Darwinian evolution, even over billions of years.

The robustness and stability of the Molecular Toolbox are demonstrated by the history of guanine. Guanine is one of the four bases of DNA and RNA. Guanine is used to not only encode genetic information in DNA, but is a primary component of ribosomes and other RNAs. Guanine is used in energy transduction and signaling. Guanine, which is endowed with remarkable capabilities for molecular recognition, is a component of the Molecular Toolbox of Life.

Around 3.7 billion years ago, when the Molecular Toolbox was established, guanine was chemically suitable as a component of genetic material and was a reasonable evolutionary choice for the Molecular Toolbox.

**Fig. 4:**
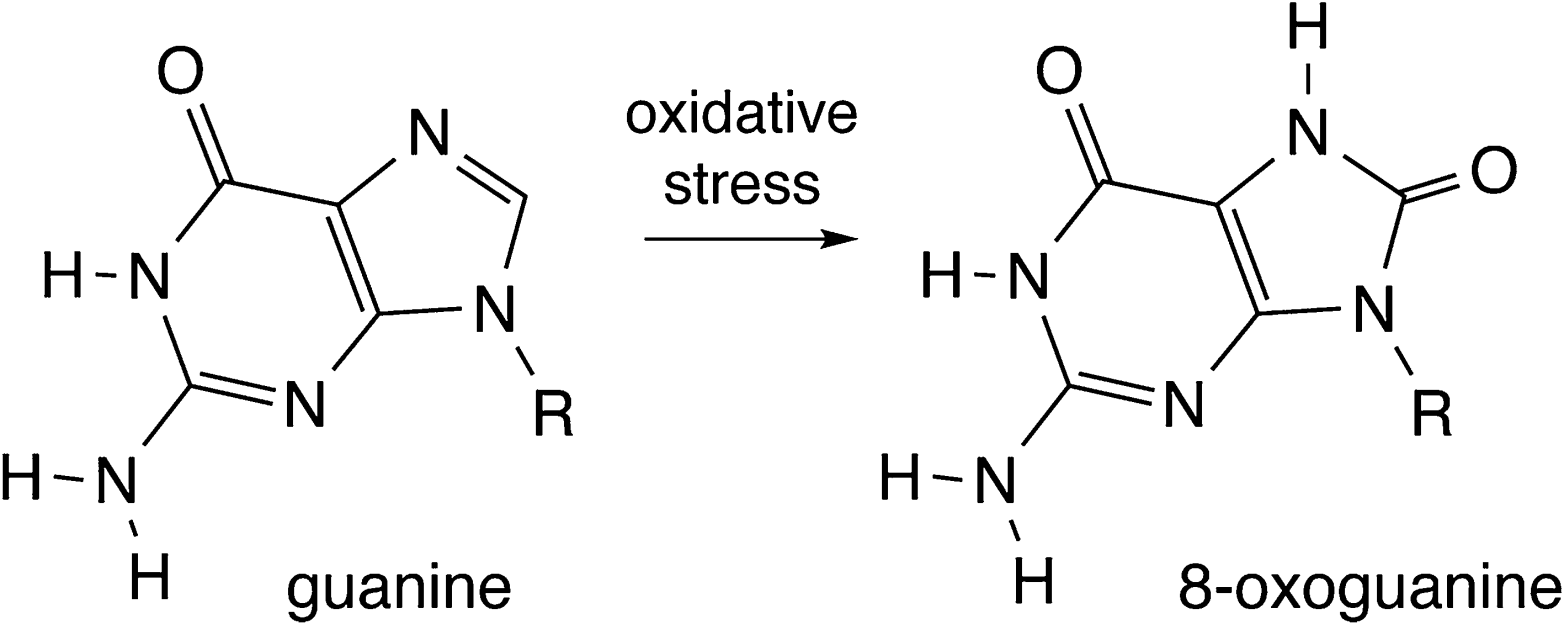
The oxidation of guanine to form 8-oxoguanine. Guanine spontaneously degrades in the oxidative environment of the post-GOE earth

However, 1.5–2.0 billion years after LUCA, with the Great Oxidation Event (GOE) (Anbar et al. 2007; Hazen et al. 2008), the oxidative potential of the biosphere changed, the chemical stability of guanine declined. Rates of guanine degradation in biological systems increased markedly. In the oxidizing environment of the extant post-GOE earth, around 100,000 guanines per mammalian cell are degraded to 8-oxoguanine each day (Fig. 4) (Grollman and Moriya 1993; Hirano 2008). A steady-state level of around one 8-oxoguanine per 10^6^ guanines is observed in mammalian cells (Delaney et al. 2012). Oxidation causes other damage, including hyperoxidized guanine. Oxidative damage to guanine in humans leads to mutagenesis, genetic instability, aging, and cancer. The inclusion of guanine in the Molecular Toolbox is a demonstration of evolution’s lack of foresight.

For the last 2 billion years, biological systems have been under intense pressure brought on by chemical instability of guanine. The permanence and integrity of genetic information and of critical energy transduction and signaling molecules are under relentless assault by oxidative processes. The pressure to change the components of the Molecular Toolbox must be intense.

What is the evolutionary response to the continuous degradation of guanine? Has evolution, over the last 2 billion years, altered the contents of the Molecular Toolbox to accommodate fundamental chemical change in the biosphere? Has evolution swapped guanine for a more appropriate substitute? No. Since the GOE, evolution has tinkered. Biology has produced elaborate and multilayered systems to repair 8-oxoguanine, and to chemically push it uphill, back to guanine (Grollman and Moriya 1993; Hirano 2008). In addition, evolution has sequestered iron and other mediators of oxidative damage (Theil and Goss 2009). As stated by Jacob (1977), “It is always a matter of using the same elements, of adjusting them, of altering here or there, of arranging various combinations…. It is always a matter of tinkering.” Evolution changes the distributions and spatial arrangements of toolbox components, but never the essential identity of the components. The persistence of guanine demonstrates that the Molecular Toolbox is fixed; alterations of the toolbox are effectively prohibited.

### Dependencies and the Limits of Evolution

A useful OOL model should account for and predict the contents and the robustness of the Universal Gene Set and the Molecular Toolbox. Why are the Universal Gene Set and Molecular Toolbox so robust over time and environment? Why is the Universal Gene Set focused on translation and not metabolism? The answers appear to be found in dependencies, which are relationships in which change in one element induces change in another element. Systems with the most extensive and far-reaching dependencies are most resistant to evolutionary change. For the Molecular Toolbox and the Universal Gene Set, biology appears to be at a limit of total dependency. The dependency of biological systems on polypeptide or polyribonucleotide, for example, is obviously complete and total. Converting polypeptide to polyester would impact every system of every cell. Converting phosphorus to arsenic in polynucleotides would also impact every system of every cell. These types of changes are not observed. The universal conservation of translation (Woese 2000; Hsiao et al. 2009) confirms the expectation that the dependencies on translation are at the same limit (Hinegardner and Engelberg 1963). Translation directly impacts all cellular functions and processes. Translation controls the sequence of amino acids of every protein. Translation is regulated by molecular interaction networks that dwarf other networks in size, integration, and evolutionary conservation (Bu et al. 2003; Butland et al. 2005). Translation consumes vast cellular resources (Warner 1999; Caton et al. 2000). Translation components are embedded in processes that appear unrelated to translation (Park et al. 2008).

## Data and the Models

### OOL Models, the Molecular Toolbox, and the Universal Gene Set

The Universal Gene Set lacks genes for the privileged functions that define conventional OOL models. The Universal Gene Set lacks genes for RNA polymerase ribozymes and metabolic ribozymes and membrane biosynthesis. Ironically, the translation system, which dominates the Universal Gene Set, is an afterthought to each of the privileged function OOL models.

Genetic and catalytic takeovers, in which the Molecular Toolbox and the Universal Gene Set are re-written, are intrinsic to RNA Worlds, Clay Worlds, and Metabolism-First Worlds. OOL models based on privileged functions necessarily depend on takeovers to transition from one genetic or catalytic system to the DNA/RNA/protein system of extant biology. In these models, Darwinian evolution drives the replacement of one Molecular Toolbox with another, and of one Universal Gene Set with another. The robustness and contents of the Toolbox of Life and the Universal Gene Set are, in these models, post hoc phenomena.

In OOL models based on privileged functions, translation initiated in a pre-existing Darwinian system, and matured after other functions to its current universality, centrality, and nexus of dependence. In these models, an entirely new type of biopolymer (coded polypeptide) was invented and implemented via Darwinian processes. New biochemistries, including biosynthesis of amino acids, charging of tRNAs, and coded protein synthesis were introduced and became essential and universal. Previous information transduction and metabolic systems were whited out of the phylogenetic record. The Molecular Toolbox was radically refurbished.

### Possible versus Plausible

There is no evidence to our knowledge that Darwinian processes can revise the Molecular Toolbox or radically alter the Universal Gene Set. Available evidence exemplified by guanine and by the robustness of the translation system suggests takeovers are unlikely by Darwinian processes.

Although privileged function OOL models with genetic and metabolic takeovers appear implausible, they are not impossible. It is conceivable that the rules and processes of Darwinian evolution have changed over time. One can imagine that in a former state of biology, the Molecular Toolbox was malleable, that the basis of the Universal Gene Set was not yet in place, and even that wholesale whiteouts of metabolic and information transduction systems were common. Although these possibilities are not consistent with evolution as we know it, they cannot be excluded.

One might consider lifting the rules, and discounting observed proscriptions because systems near the OOL were pre-Darwinian. However, the putative RNA World, for example, is explicitly a Darwinian environment. In fact, one can say that a primary motivating rationale for RNA World models is to initiate Darwinian processes as early as possible relative to the OOL.

### A Conservative Approach to the OOL

A primary attraction of conventional OOL models and their privileged functions is an appearance of simplicity. These models allow one to consider replication only, or metabolism only, or chemical gradients only as dominant phenomena. However, the simplicity of these models is seen to be an illusion on the realization that the models require fluidity in principles of evolution. These models invoke genetic takeovers and toolbox replacements, which are required for transitions from privileged function worlds to extant biology. In fact, once the takeover processes are acknowledged and are reasonably considered, the privileged function models are seen to be extremely complex with poor predictive power; they require indeterminacy and plasticity in the rulebook that governs biological processes.

Occam’s razor, which states that the simplest hypothesis is most probable, supports a model in which the Universal Gene Set and Molecular Toolbox were robust not only post-LUCA but pre-LUCA and were not re-invented by Darwinian processes, and that genetic and catalytic takeovers are unlikely. In this scenario, RNA, DNA, and protein and the Molecular Toolbox co-evolved in a cooperative and symbiotic process, with joint participation of many molecular participants and processes during the OOL and during the conversion from chemical to biological evolution. In this scenario, there were no privileged functions. If these conservative assumptions are correct, then conventional models of the OOL, with their privileged functions, require reconsideration.
